# Virtual Fracture Clinics: Are They Truly Safe and Cost-Effective?

**DOI:** 10.7759/cureus.99898

**Published:** 2025-12-22

**Authors:** Sunandan Datta, Mahak Baid, Bratati Bandyopadhyay, Mohammed Hussain

**Affiliations:** 1 Trauma and Orthopaedics, Aneurin Bevan University Health Board, Newport, GBR

**Keywords:** face-to-face fracture clinic, fracture-clinic, orthopaedic clinic, vfc, virtual fracture clinic

## Abstract

The face-to-face traditional fracture clinic (TFC) model for managing acute musculoskeletal trauma in the UK has faced increasing criticism for being inefficient, costly, and often failing to meet timely specialist review standards. In response, the virtual fracture clinic (VFC) model was introduced, replacing automatic physical appointments with a consultant-led virtual review of clinical notes and radiographs to appropriately triage patients. This narrative review examines the current evidence on the safety, efficacy, and cost-effectiveness of this virtual approach. Evidence strongly supports the VFC model's safety, with systematic reviews covering over 63,000 patients reporting low re-attendance rates (under 5%) and minimal missed diagnoses, comparable to or better than TFCs. The VFC structure enhances clinical governance, routinely achieving compliance rates above 83% for the national standard of specialist review within 72 hours, a significant improvement over the performance of many traditional clinics. Furthermore, the economic case is robust, with VFCs consistently demonstrating substantial cost savings for healthcare systems by reducing unnecessary appointments and optimizing consultant time. Patient satisfaction is generally high, primarily due to convenience, though ensuring high-quality, structured communication and robust safety-netting is essential to mitigate concerns related to the lack of physical examination. Overall, VFCs provide a safe, efficient, and cost-effective method for managing selected injuries, serving as a critical complementary tool that reserves physical clinic capacity for patients with complex needs.

## Introduction and background

The management of acute musculoskeletal trauma in the UK has traditionally relied on the fracture clinic model, a mainstay of orthopaedic practice for decades. In this traditional system, patients presenting to the emergency department (ED) with suspected fractures are typically referred to a consultant-led, face-to-face traditional fracture clinic (TFC) for review. However, this approach has increasingly faced criticism for being inefficient, overburdened, and often inconvenient for patients. As patient volumes rise and healthcare budgets tighten, these inefficiencies can lead to overcrowded waiting rooms, delayed reviews, and variable adherence to recommended guidelines [1-3]. 

In 2011, the Glasgow Fracture Pathway introduced an alternative virtual fracture clinic (VFC) model with the potential to reduce traditional clinic visits, waiting times, and overall costs. The VFC fundamentally restructures the patient pathway: instead of an automatic physical appointment, clinical notes and radiographs are reviewed virtually by a consultant orthopaedic surgeon. Following this review, patients are triaged into one of several streams: direct discharge with advice, referral to a specialist clinic, urgent surgical review, or a routine face-to-face appointment [4,5]. 

The adoption of VFCs has been accelerated by two major factors: the British Orthopaedic Association Standards for Trauma (BOAST) guidelines, which mandate a fracture clinic review within 72 hours of presentation, and the COVID-19 pandemic, which necessitated a rapid reduction in unnecessary hospital footfall. While the theoretical benefits of VFCs-speed, efficiency, and cost savings-are compelling, concerns remain regarding their safety. Does removing the physical examination from the initial specialist review increase the risk of missed diagnoses? Do patients feel inappropriately managed without a formal clinical assessment? Many units have now implemented this model in the non-operative management of simple, undisplaced fractures [2,6,7]. 

This narrative review examines the current evidence regarding the safety, clinical efficacy, and cost-effectiveness of VFCs.

## Review

Safety and clinical efficacy 

The primary concern accompanying the shift to virtual triage is safety. Critics of the VFC model initially feared that the absence of a physical examination would lead to missed injuries, inappropriate discharges, and ultimately poor functional outcomes. However, literature from the past decade, including systematic reviews published as recently as 2025, largely allays these fears [6-8]. 

A critical metric for safety is the rate of missed diagnoses, patients discharged who actually required intervention. A systematic review by Johnson et al. assessed data from over 63,000 patients and found that VFCs have a re-attendance rate of less than 5% following discharge. Reported false-negative rates are as low as 4%, and some cohorts have documented zero missed fractures during their study periods [5,7]. 

The “safety-netting” built into the VFC model appears to be a key protective factor. In a standard VFC workflow, if a patient’s symptoms do not align with the diagnosis (e.g., persistent pain in a presumed sprain), open-access return policies allow them to re-enter the system easily. Research indicates that unexpected re-presentation rates are low, typically between 4% and 7.5%. Most re-attendances are related to pain management or minor concerns rather than serious missed injuries. A retrospective analysis of 17,269 referrals by Dey et al. in a UK trust found that although 7.48% of virtually discharged patients re-presented, 98% were managed conservatively, and fewer than 0.2% required unanticipated surgery [9]. 

One of the strongest arguments for the clinical efficacy of VFCs is their ability to meet national standards. The British Orthopaedic Association Standards for Trauma guidelines require that patients with acute traumatic injuries be reviewed by a specialist team within 72 hours. Traditional clinics, often operating at capacity, frequently fail to meet this benchmark, with compliance rates documented as low as 5.7%. In contrast, VFCs routinely demonstrate compliance rates above 83%, with some trusts achieving 100%. By decoupling review from the availability of physical clinic slots, consultants can assess large volumes of cases rapidly, ensuring timely identification and prioritisation of significant injuries [2,7,10]. 

While the model is broadly safe, the literature suggests that not all injuries are equally suited to virtual management. “Stable” injuries-such as Weber A ankle fractures, clavicle fractures, paediatric torus fractures, and 5th metatarsal fractures-show high success rates with direct discharge protocols [11-13]. 

Cost-effectiveness 

The economic case for VFCs is among the most robustly supported findings in the literature. TFCs are resource-intensive, requiring physical space, nursing staff, administrative support, and significant consultant time per patient. 

Financial analyses consistently demonstrate substantial savings for healthcare providers. An interrupted time series analysis found that implementing a VFC saved a local Clinical Commissioning Group (CCG) approximately £129,885 annually. Larger trusts have reported even greater efficiencies; one analysis of more than 17,000 referrals estimated annual savings of £702,205. Anderson et al. reported a mean cost per patient of £22.84 in the VFC pathway compared with £36.81 in the TFC pathway [9,14,15]. 

These savings arise from several sources. VFCs typically reduce face-to-face clinic volumes by 50-65%, allowing hospitals to redeploy clinic space and staff. They also improve staffing efficiency, as consultants can review virtual cases in approximately two to three minutes compared with the 10-15 minutes required for in-person consultations, enabling senior clinicians to oversee significantly larger caseloads. In addition, standardised imaging protocols within VFC pathways help minimise unnecessary repeat radiographs that are more likely to occur in traditional models [14-16]. 

Beyond hospital savings, VFCs also reduce societal costs. Under the traditional model, a patient with a minor fracture may need to take time off work, travel to the hospital, and incur parking fees, only to receive simple advice. In a VFC system, this information is delivered remotely, often via phone call or digital information pack. Although societal savings are harder to quantify precisely, reductions in travel, lost productivity, and waiting times are widely acknowledged [14,15,17]. 

Patient satisfaction and experience 

While safety and cost are quantifiable metrics, patient satisfaction is more subjective and multifaceted. Overall, satisfaction with VFCs is high, though not universal. Multiple studies report patient satisfaction rates between 75% and 97%. Convenience is the most frequently cited benefit: patients value avoiding unnecessary travel and receiving timely diagnoses and management plans. A systematic review reported a mean satisfaction rate of 85.4%, with many patients expressing a preference for virtual review over traditional clinic attendance [7,15]. 

A consistent minority of patients report dissatisfaction, often related to communication. Some feel that virtual consultations lack the reassurance of a physical examination. Others report that phone calls feel “too quick” or that they were unable to ask questions comfortably. In one study, while 75% were satisfied, 25% later attended in person because they “could not get the full message over the phone” or had difficulty hearing [16-18]. 

In paediatric settings, satisfaction among parents is generally high, particularly due to the convenience of avoiding hospital visits with children. However, parental anxiety remains a common driver of re-attendance. Studies show that when parents receive clear red-flag guidance and an open-return policy, their confidence increases, and satisfaction rates approach or exceed those of traditional clinics [19-21]. 

Limitations of the VFC pathway 

The quality of information provided to patients is critical in VFC pathways. Models that incorporate structured written or digital materials, such as standardised leaflets, exercise guides, and clear rehabilitation timelines, report higher satisfaction and lower unplanned re-attendance. This mirrors broader musculoskeletal telehealth literature, where multimodal education (written materials plus videos) improves engagement and reassurance compared with verbal advice alone. Conversely, telemedicine models relying on brief, one-off remote contacts without supporting written information can leave patients feeling uncertain or anxious about their diagnosis and self-management. The potential depersonalisation of care is a recurring theme, particularly for older adults and individuals with lower health or digital literacy, who may find it challenging to voice concerns or navigate remote systems [18-21]. 

Discussion 

The transition from TFCs to VFCs represents a paradigm shift in orthopaedic trauma management, driven by necessity and increasingly supported by evidence. Our review suggests that VFCs are safe and efficient for the vast majority of patients. Missed diagnosis rates are comparable to, or even better than, those reported in traditional clinics, largely because every X-ray is reviewed by a consultant, a key distinction from the conventional face-to-face model. The overall safety of the system relies heavily on robust safety-netting procedures and the ability for patients to return easily if symptoms persist or worsen [16,20]. 

In terms of cost-effectiveness, the evidence is compelling. VFCs generate substantial financial savings for healthcare systems by reducing unnecessary appointments and optimising consultant time, all without compromising outcomes for standard trauma presentations. However, the virtual model is not universally applicable. It depends on a well-developed infrastructure of digital communication tools, high-quality patient education resources, and responsive administrative support. VFCs perform best when applied to clearly defined, protocol-driven injuries and are less suitable for complex presentations requiring nuanced assessment or physical examination. Additionally, although patient satisfaction is generally high, it is strongly influenced by the quality of communication; an effective VFC should prioritise active, patient-centred engagement rather than one-way information delivery [9,21-23]. 

An additional consideration is the potential medico-legal impact of increased reliance on virtual decision-making. Although VFCs demonstrate low rates of missed injuries, the reduced opportunity for physical assessment may heighten clinicians' perceived liability, particularly in cases where subtle clinical signs, such as ligamentous laxity or neurovascular compromise, are difficult to appreciate from radiographs alone. Clear documentation, structured triage algorithms, and robust escalation pathways are therefore essential for mitigating risk. Furthermore, future research should explore long-term functional outcomes, as much of the current evidence focuses on short-term safety and service efficiency. Understanding how virtual care affects recovery trajectories, adherence to rehabilitation, and health equity will be critical as VFC models continue to expand across diverse healthcare settings [6,7].

## Conclusions

VFCs provide a safe, efficient, and cost-effective alternative to TFCs for the management of selected musculoskeletal injuries. Ensuring high-quality communication, structured patient education, and adequate safety‑netting is essential for sustaining patient satisfaction and clinical safety. VFCs should be viewed as a complementary tool that enhances, rather than replaces, the face-to-face fracture clinic, so that it is reserved for only those who need it the most. 
